# Phosphorus fractionation and distribution in Nitisols of the central Ethiopian highlands affects soil nutrient management strategies in barley (*Hordeum vulgare* L.) production

**DOI:** 10.7717/peerj.20410

**Published:** 2025-12-19

**Authors:** Matiyas Dejene Girma, Miheretu Bedassa Wakessa

**Affiliations:** Soil and Water Management, Ethiopian Institute of Agricultural Research, Addis Ababa, Ethiopia

**Keywords:** Occluded P, Aluminum bonded, Iron bonded, Inorganic phosphorus fraction, Soil acidity

## Abstract

Optimizing nutrient management is essential for sustaining crop productivity on fertilizer-unresponsive acidic soils, particularly through a comprehensive understanding of soil phosphorus (P) dynamics. Phosphorus availability is often limited by soil physicochemical properties, necessitating site-specific amendment strategies. This study evaluated the status of inorganic P fractions and their response to various nutrient and soil amendment treatments in acidic Nitisols from Welmera and Ejere districts in the central Ethiopian highlands, with a focus on barley (*Hordeum vulgare*), a crop that is sensitive to low soil pH. Field and laboratory investigations revealed that strongly acidic soils responded positively to lime application combined with optimal P fertilization, resulting in significant yield improvements. In contrast, moderately to slightly acidic soils exhibited limited response to liming but showed enhanced productivity following organic amendments such as compost. Soil management strategies including the application of inorganic phosphorus fertilizers, increased barley yield to 3479 kg ha^−1^. Additionally, soil amendments such as agricultural lime improved barley productivity by enhancing the availability of plant-accessible phosphorus. Sequential phosphorus fractionation revealed that iron-bound and occluded P fractions dominated the inorganic P pool, accounting for approximately 45% and 35% of total inorganic P, respectively. Higher level of aluminum-bound P was also observed in strongly acidic soils. higher water-soluble P represented only about 3% of total inorganic P, highlighting the limited readily available P fraction. These findings emphasize the critical role of detailed P fractionation analysis in informing targeted nutrient management strategies to improve P availability, thereby supporting sustainable soil fertility and crop production, particularly barley under acidic soil conditions.

## Introduction

Soil fertility degradation is a major constraint limiting agricultural productivity globally, driven by factors such as population growth, climate change, and soil erosion. In East Africa, decades of nutrient mining and soil erosion have exacerbated land degradation, leading to widespread nutrient deficiencies (Mulinge et al., 2016). Among these, nitrogen (N) and phosphorus (P) deficiencies are particularly critical, severely limiting crop productivity in the region (Kihara et al., 2016; Kiwia et al., 2019; Mutegi et al., 2018; Sileshi et al., 2019).

Phosphorus limitation is especially severe in areas where parent materials inherently contain low P levels or in soils with high P-retention capacities, such as acidic or calcareous soils (Batjes, 2011). In Ethiopia, many farmlands fail to achieve expected crop yield responses due to soil physicochemical constraints, including acidity, alkalinity, waterlogging, and salinity. For instance, acidic soils in regions like Welmera and Farta districts immobilize phosphorus by forming insoluble compounds with aluminum and iron oxides, reducing the availability of applied fertilizers (Kiflu, Beyene & Jeff, 2017; Tadesse, Alemayehu & Abebe, 2019). Similarly, waterlogged and saline soils exacerbate nutrient imbalances, further limiting crop productivity (Gebreyesus, Mamo & Tesfaye, 2018; Desta & Abebe, 2020).

Despite the critical role of phosphorus in crop production, most smallholder farmers in East Africa apply fertilizers at rates far below recommended levels due to economic constraints and limited access to inputs (Duflo, Kremer & Robinson, 2008; Kiwia et al., 2019; Sileshi et al., 2019). This insufficient application of fertilizers is compounded by a lack of information on the source–sink dynamics of applied phosphorus across diverse soil types. Minimal application rates of phosphorus in diverse soils are challenging to set due to varying source–sink dynamics, including how phosphorus is added, transformed, fixed, or lost in soils with different chemistry, texture, pH, and biological activity without precise knowledge of these dynamics, fertilizers may be under-applied, leading to yield loss, or over-applied, causing environmental runoff. Efficient fertilizer P use depends on 4R nutrient stewardship: applying the right fertilizer source, at the right rate, at the right time, and in the right place (Roberts & Johnston, 2015) so its management focuses on minimizing application while ensuring sufficient supply, tailored to soil-specific behaviors to reduce losses and optimize crop uptake (Riemersma et al., 2006; Katrina et al., 2018). Understanding these dynamics is essential for improving nutrient use efficiency and developing effective input management strategies to enhance crop productivity while maintaining soil fertility.

Phosphorus fractionation studies have been instrumental in understanding the transformation and availability of phosphorus in soils under different management practices. The concept of dynamic transfer among P pools underpins understanding of P availability, and informs fertilizer recommendations to optimize crop uptake while maintaining soil fertility (Roberts & Johnston, 2015). The fractionation method developed by Chang & Jackson (1957) has been widely used to investigate inorganic phosphorus forms and transformations, which involve sequentially extracting phosphorus from soil using different chemical extractants at each step to separate phosphorus into distinct pools based on their availability and chemical form within the soil matrix. However, subsequent research has highlighted limitations in its specificity for certain soil types (Fife, 1962; Petersen & Corey, 1966; Williams, Payne & Syers, 1967; Smillie & Syers, 1972). Modifications to this method have improved its applicability across various soil environments.

In Ethiopia’s acidic soils, P availability is a critical limiting factor for crop productivity due to the strong fixation of phosphorus by Al and Fe compounds under low pH conditions. Integrated soil management approaches such as liming and organic amendments have shown considerable promise in enhancing phosphorus availability and thus improving crop yields (Kiflu, Beyene & Jeff, 2017; Tadesse, Alemayehu & Abebe, 2019). Liming raises soil pH by neutralizing acidity, which in turn reduces aluminum toxicity and the sorption of phosphorus, thereby increasing the concentration of plant-available phosphorus fractions (Regasa, Haile & Worku, 2025; Yigezu, Gebresilassie & Abate, 2023). Organic amendments like vermicompost and biochar also contribute by improving soil structure, biological activity, and nutrient cycling, further enhancing phosphorus mobilization and uptake by crops (Regasa, Haile & Worku, 2025; Oumer, Kassahun & Jibat, 2024).

Phosphorus fractionation studies in Ethiopian acid soils reveal that available P often exists in low concentrations due to fixation in less labile inorganic fractions associated with Al and Fe oxides (Chulo et al., 2022; Yigezu, Gebresilassie & Abate, 2023). Liming not only increases soil pH but also shifts the distribution of phosphorus from less available forms to more labile and bioavailable fractions, facilitating higher phosphorus uptake by plants (Ejigu, Mengistie & Wassie, 2023). This effect is often amplified when lime is combined with phosphorus fertilization and organic inputs, resulting in significant improvements in crop growth and yield (Regasa, Haile & Worku, 2025; Tadesse, Alemayehu & Abebe, 2019). For staple crops like maize and barley, studies from southwestern and central Ethiopian highlands show that integrated application of lime and organic amendments along with phosphorus fertilizers can result in 38–78% yield increases compared to non-amended acid soils (Regasa, Haile & Worku, 2025). Moreover, the enhanced phosphorus availability not only supports grain development but also improves overall nutrient use efficiency, root development, and crop resilience to abiotic stresses typical in acidic agro-ecologies (Regasa, Haile & Worku, 2025; Kiflu, Beyene & Jeff, 2017). Therefore, phosphorus fractionation studies and their application through integrated soil fertility management are critical in addressing nutrient limitations in Ethiopia’s acid soils. These strategies contribute to sustainable agricultural intensification by improving soil chemical properties, increasing phosphorus bioavailability, and ultimately enhancing crop productivity under challenging soil acidity conditions. A study by Agegnehu & Lakew (2013) determined the optimum P rate in the study area which investigated the minimum phosphorus level for optimal barley yield in the Ethiopian highlands is a soil test P of 12 mg kg^−1^, and a phosphorus fertilizer addition of about 30 kg P per hectare is optimal for significant yield improvements, with maintenance rates recommended at 10 kg P per hectare annually to sustain soil fertility and yield. These findings underscore the need for further research on phosphorus fractionation to optimize fertilizer use in diverse agroecological contexts. Therefore, this study aims to address existing knowledge gaps by investigating phosphorus dynamics and providing insights into the effective utilization of non-renewable resources in an environmentally sustainable manner.

## Objectives

• To compare and contrast different inorganic phosphorus pools in Nitisols of central Ethiopian highland and their relation with different soil amendments with phosphorus fertilizer application
• To investigate the status of applied phosphorus fertilizer with other forms of soil inorganic phosphorus pools
• To evaluate the interaction effect of soil amendments with phosphorus fertilizer application on the agronomic performance of food barley.

## Material and method

The field and laboratory experiments were conducted between 2022 and 2024 in Welmera (Holeta and Sademo kebeles) and Ejere districts (Damotu PAs) of the central Ethiopian highlands. The sites were selected based on variations in soil pH, acidity, and diverse physico-chemical properties to represent a range of Nitisol soils from strongly to slightly acidic within the potential barley-producing areas of the central Ethiopian highlands. These Nitisols are characterized by deep, well-drained profiles with a clay subsurface horizon exhibiting typical blocky structure and shiny ped faces. They commonly have a pH range of 4.5 to 6.5, high clay content (often exceeding 40%), moderate to strong acidity, and significant phosphorus fixation capacity. The soils also exhibit good internal drainage, fair water-holding capacity, and considerable cation exchange capacity due to their clay and low organic matter content. The study areas receive an average annual rainfall of 1,100 mm and have yearly mean maximum and minimum temperatures of 22.2 °C and 16.1 °C, respectively, based on 10-year (2013–2023) averages from the HARC meteorological station.

Food barley (variety BH-1307) which was sourced from the HARC barley breeding program was used as the test crop during the 2022 main cropping season (Meher), with the total growth duration from June 10 to November 27, when the crop was harvested. The experiment employed a factorial randomized complete block design (RCBD) with three replications, with a plot size of 12 m^2^ or 3 by 4 m length and width respectively. The treatments consisted of a factorial combination of three phosphorus levels and three lime application rates, resulting in a total of 27 experimental units per site. These treatments were replicated across three different sites, bringing the total number of experimental units to 81:

• P1 = Recommended phosphorus from triple superphosphate (TSP) at 30 kg P ha^−^^1^ rate which composed of 46% P_2_O_5_
• P2 = Recommended phosphorus rate from compost based on the nutrient test, and
• P3 = No phosphorus input.

Additionally, three lime application levels were tested:

• LM1 = Full lime rate based on soil exchangeable acidity (EA) result, (*i.e.,* 433, 285, and 2,104 kg ha^−^^1^ for the HARC, Sademo, and Damotu study sites, respectively)
• LM2 = one-fourth of the full lime rate, and
• LM3 = no lime application.

Before the experiment, composite soil samples were collected from the three study sites (Sademo, Holeta, and Damotu) and analyzed for exchangeable acidity at the Holeta Agricultural Research Center (HARC) soil and plant analysis laboratory. The required lime application rates were determined using the exchangeable acidity method (EA) described by Kamprath (1984). Lime was applied based on the treatment specifications: LM1 received the full dose of lime determined from EA, LM2 received one-fourth of the rate determined from EA, and LM3 received no lime application. Agricultural lime (CaCO_3_) was used as the source of lime, it was applied four weeks prior to planting at rates of 433, 285, and 2,104 kg ha^−^^1^ for the HARC, Sademo, and Damotu sites, respectively. These application rates were calculated based on the exchangeable acidity values measured at each site (HARC: 0.35 meq/100 g soil, pH 5.0; Sademo: 0.23 meq/100 g soil, pH 5.4; Damotu: 1.7 meq/100 g soil), which correspond to the first lime treatment rate (LM1). A second lime treatment rate, LM2, was set at one-quarter of the recommended rates, equivalent to 107, 71.2, and 526 kg ha^−^^1^ for the respective experimental sites. The phosphorus fertilizer treatments consisted of an inorganic source, triple superphosphate (TSP) chemical formula is Ca(H_2_PO_4_)_2_⋅H_2_O (monocalcium phosphate monohydrate) in its fertilizer form. Which have 46% P_2_O_5_ (phosphorus pentoxide) for fertilizer-grade TSP and manufactured from OCP Group (Morocco), and an organic source, conventional compost prepared locally, represented as P1 and P2, respectively, while P3 received no phosphorus fertilizer. The application rate of the organic fertilizer was determined based on the available phosphorus content in the compost which was determined by laboratory test prior to the application accordingly the compost (0.7% available phosphorus, 25% moisture, and 0.86% total nitrogen). Thus, a 5,700 kg ha^−^^1^ compost rate was applied as recommended fertilizer rate for treatment P2. The recommended rate of inorganic phosphorus for the study area and crop type was 30 kg P ha^−1^ or 69 kg P_2_O_5_ per hectare (Agegnehu & Lakew, 2013), equivalent to 150 kg TSP per hectare. Both phosphorus sources were applied at planting. Additionally, a recommended nitrogen rate of 60 kg N per hectare was applied to all treatments using urea fertilizer, split into two applications: the first at planting and the second at the tillering growth stage of the barley crop. All other agronomic practices were carried out according to standard recommendations.

## Soil Sample collection and analysis

Soil samples were collected at three distinct barley growth stages. (1) The first sampling occurred before planting, where composite soil samples were taken for characterization purposes. (2) The second sampling was conducted at three weeks after planting, with soil collected from each experimental plot to analyze inorganic phosphorus fractionation. and (3) The third and final soil sample was taken at the harvesting stage to assess the residual nutrient status of the various treatment applications. Soil pH was measured using a 1:2.5 soil-to-water (H_2_O) ratio; Total nitrogen (TN) was determined using the Kjeldahl method (Bremner, 1960); Available phosphorus (AP) was tested by using Bray-II method in ppm (Bray & Kurtz, 1945), and organic carbon (OC) was measured using the Walkley & Black (1954) method. Exchangeable acidity (EA) was determined by using the KCl method and determined in cmol (+)/kg (HJ, 2013). Exchangeable cations and cation-exchange capacity (CEC) were determined by using the ammonium acetate method (Black, 1965) and expressed in cmol (+)/kg. inorganic phosphorus fractionation was conducted by using Chang & Jackson (1957) sequential phosphorus extraction procedure.

## Sequential extraction procedure of inorganic Phosphorus

**Figure 1:**
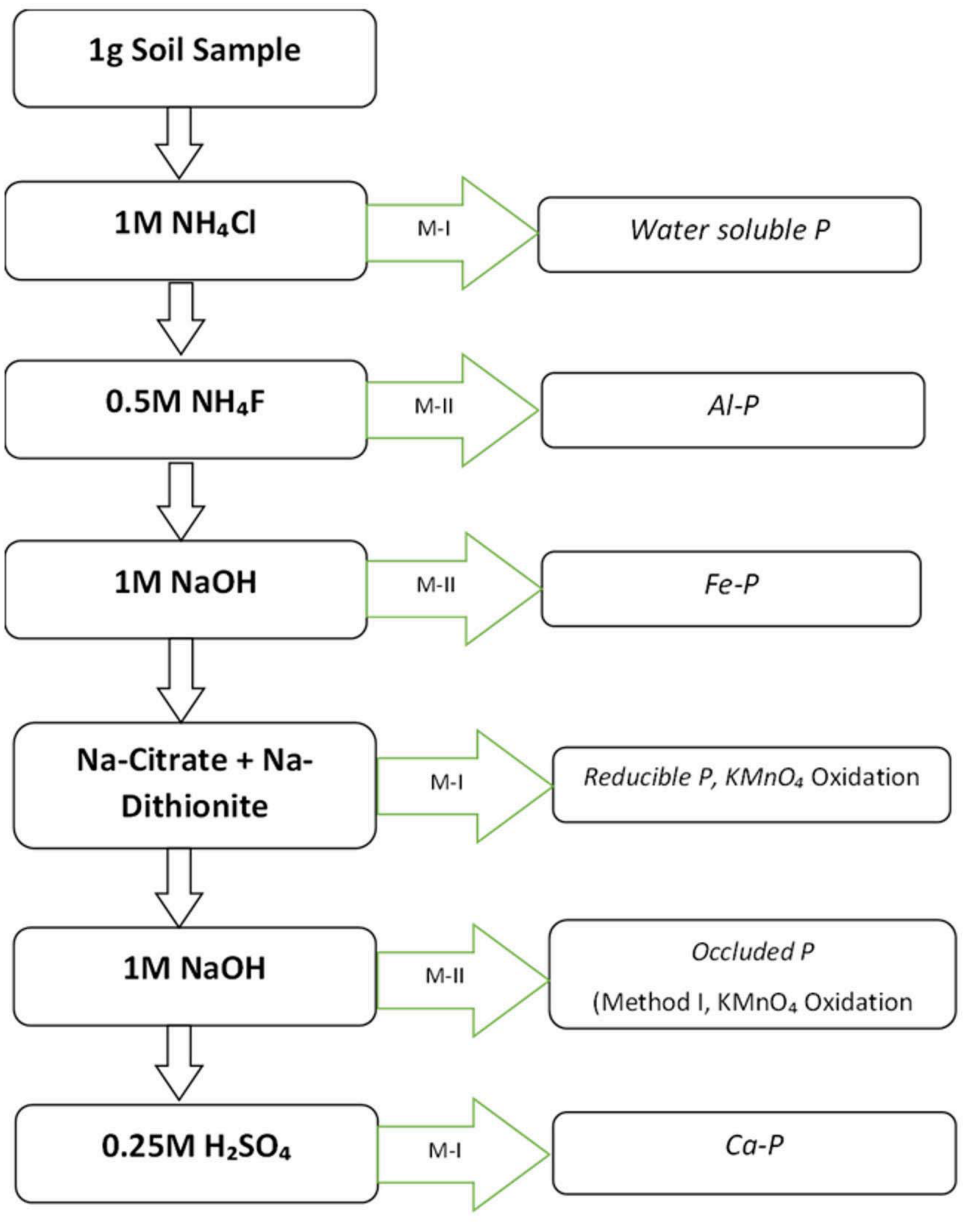
Schematic work flow for sequential soil inorganic P fractionation (Chang & Jackson (1957). M-I and M-II refers method of P determination as described in the Supplemental File.

A single 1 g soil sample (0–15 mm fraction) was subjected to a sequential phosphorus (P) extraction process using different chemical reagents to isolate specific P fractions (Chang & Jackson, 1957). Each extraction step was performed on the same soil residue remaining from the previous step, ensuring a sequential separation of phosphorus fractions (Fig. 1).

Initially, 1 g of soil sample was placed in a polypropylene centrifuge tube, and 50 mL of 1M ammonium chloride (NH_4_Cl) solution was added. The mixture was shaken for 30 min and then centrifuged. The supernatant obtained was analyzed for solid-bonded phosphorus using the chlorostannous acid in sulfuric acid method (Pons and Guthrie, Method I (Supplemental Information 1).

Following this, the soil residue from the first extraction was retained, and 50 mL of 0.5M ammonium fluoride (NH_4_F) solution, adjusted to pH 8.2 with ammonium hydroxide (NH_4_OH), was added. The sample was shaken for one hour and centrifuged. The supernatant was filtered through activated carbon, and aluminum-bonded phosphorus (Al-P) was determined using Method II, which involves chlorostannous acid in hydrochloric acid (Supplemental Information 2).

Next, the soil residue was washed twice with 25 mL of saturated sodium chloride (6M NaCl) solution which is prepared by dissolving 359g of NaCl in one liter of distilled water, centrifuging after each wash and discarding the washings. Then, 50 mL of 1M sodium hydroxide (NaOH) solution was added, and the mixture was shaken for 17 h before centrifugation. The supernatant was treated with five drops of concentrated sulfuric acid (H_2_SO_4_), centrifuged again, and filtered through 0.5 g of activated carbon. Iron-bonded phosphorus (Fe-P) was determined using Method II (Supplemental Information 2).

The soil residue was washed twice more with saturated 6M NaCl solution, discarding the washings. It was then suspended in 25 mL of 0.3M sodium citrate solution, followed by the addition of 1 g of sodium dithionite. The mixture was shaken for 15 min, heated to 80 °C, diluted to 50 mL, shaken for an additional 5 min, and centrifuged. The supernatant and washings were combined, and 1.5 mL of 0.25M potassium permanganate (KMnO_4_) was added to oxidize excess dithionite and citrate. After standing for 2 min, reductant-soluble phosphorus was determined using Method I. Subsequently, the soil residue was washed twice with saturated 6M NaCl solution, and the washings were combined with the previous extract. Then, 50 mL of 0.1M NaOH solution was added, and the sample was shaken for one hour before centrifugation. Occluded phosphorus was determined using Method II (Supplemental Information 2).

Finally, the soil residue was washed twice with saturated 6M NaCl solution, and 50 mL of 0.25M sulfuric acid (H_2_SO_4_) was added. The mixture was shaken for one hour and centrifuged. Calcium-bonded phosphorus (Ca-P) was determined using Method I.

## Data collection and analysis

During the field experiment, comprehensive agronomic data were collected to evaluate the effects of treatments on barley growth and productivity at physiological maturity stage of barley after which no more growth parameter data change was is expected. Growth parameters measured included plant height, spike length, and the number of tillers per plant. Yield components such as above-ground dry biomass, grain yield, thousand-seed weight, hectoliter weight, and grain moisture content were also recorded to provide a detailed assessment of crop performance after the crop was harvested. Harvest Index (HI) is calculated as the ratio of the economic yield (usually grain or seed yield) to the total above-ground biomass produced by the barley.

Harvest Index (HI) = (Grain Yield/Total Above-ground Biomass) *100%

where both grain yield and total biomass are expressed on a dry weight basis.

It is a critical measure used in agriculture to assess the efficiency with which a crop converts assimilated photosynthates into the harvestable product. It indicates the partitioning of biomass between the grain (or other economic yield) and the total plant biomass. A higher harvest index reflects a crop’s better ability to allocate resources toward the harvestable part, thus indicating greater yield potential.

For data analysis, statistical procedures were conducted to determine the significance of treatment effects on key dependent variables including grain yield, biomass yield, HI, and thousand seed weight (TSW). Since both lime and phosphorus treatments were applied, a two-way analysis of variance (ANOVA) was employed to test the main effects as well as the interaction between these factors. When significant differences were detected, means were separated using Least Significant Difference (LSD) test at a 5% significance level (α = 0.05) to evaluate the statistical relationships among all treatment groups.

Additionally, when multiple dependent variables were analyzed simultaneously and likely correlated, a multivariate analysis of variance (MANOVA) was conducted to account for these correlations appropriately. The assumptions of ANOVA, including homogeneity of variances and normality of residuals, were checked using Levene’s test and the Shapiro–Wilk test, respectively. In cases where these assumptions were violated, data transformations (such as log transformation) were applied, or non-parametric alternatives were considered where appropriate.

All statistical analyses were performed using R statistical software version 4.2.2. This approach allowed for a rigorous and comprehensive evaluation of the effects of phosphorus and lime treatments on barley growth and yield characteristics under the specific agroecological conditions of the study sites.

**Table 1:** Barley GYD, DBM, TSW and HI summary ANOVA table for the application of lime and different phosphorus fertilizer.

**Source**	**Df**	**Sum Sq**	**Mean Sq**	**F value**	**Pr(>F)**	**Sig.**
LM	2	808,042	404,021	1.797	0.175908	
P	2	7023,111	3511,556	15.620	4.868e−06	***
LON	2	1,9019,435	9509,718	42.302	1.242e−11	***
REP	2	648,748	324,374	1.443	0.245544	ns
LM*P	4	1296,785	324,196	1.442	0.233334	ns
LM*LON	4	3365,659	841,415	3.743	0.009468	**
P*LON	4	620,742	155,185	0.690	0.601985	ns
LM*P*LON	8	1385,558	173,195	0.770	0.630168	ns
GYD_CV (%)	15.9					
LM	2	1,2144,124	6072,062	3.9	0.0264	*
PS	2	2,9144,666	1,4572,333	9.36	0.0003	**
REP	2	3309,312	1654,656	1.06	0.3528	ns
LON	2	1.51E+08	7,5439,710	48.46	<.0001	***
LM*LON	4	1,3087,311	3271,828	2.1	0.0938	ns
PS*LON	4	2104,797	526,199.2	0.34	0.8511	ns
LM*PS*LON	12	1,8518,074	1543,173	0.99	0.4698	ns
DBM_CV (%)	15.7					
LM	2	86.91062	43.45531	6.44	0.0032	**
PS	2	10.9521	5.476049	0.81	0.4495	ns
REP	2	0.617284	0.308642	0.05	0.9553	ns
LON	2	525.4647	262.7323	38.96	<.0001	***
LM*LON	4	37.98123	9.495309	1.41	0.2443	ns
PS*LON	4	23.8242	5.956049	0.88	0.4805	ns
LM*PS*LON	12	97.00148	8.083457	1.2	0.309	ns
TSW_CV (%)	6.05					
LM	2	155.9602	77.98012	4.71	0.0132	*
PS	2	89.80543	44.90272	2.71	0.0759	ns
REP	2	40.06247	20.03124	1.21	0.3067	ns
LON	2	2,364.226	1,182.113	71.36	<.0001	***
LM*LON	4	124.6435	31.16086	1.88	0.1277	ns
PS*LON	4	48.97827	12.24457	0.74	0.5696	ns
LM*PS*LON	12	226.7585	18.89654	1.14	0.3495	ns
DBM_HI (%)	10.7					

**Notes.**

Where: GYD, grain yield; DBM, dry biomass; TSW, thousand seed weight; HI, harvest index; LM, lime treatments; P, phosphorus fertilizer; LON, Locations; REP, Replication; ns, not significant. Df, degree of freedom.

ns Not significant (*p* > 0.05).

* Significant at *p* < 0.05.

** Highly significant at *p* < 0.01.

*** Very highly significant at *p* < 0.001 at the 5% probability level.

## Results and Discussion

As shown in Table 1 phosphorus treatment (P) and location (LON) had highly significant effects on the measured variable (*P* < 0.001). Additionally, the interaction between lime application (LM) and location (LM*LON) was significant (*P* = 0.009). In contrast, lime application treatment alone (LM), replication (REP), and other interaction terms (LM*P, P*LON, LM*P*LON) did not show significant effects (*P* > 0.05). The initial soil analysis indicated significant variability in acidity levels across the study sites, which necessitated the application of varying lime rates tailored to these differences. Soils with higher acidity were treated with proportionally greater lime quantities to effectively neutralize soil acidity and optimize pH levels, thereby enhancing nutrient availability and promoting crop growth. This site-specific liming approach was critical in improving soil chemical properties, particularly phosphorus availability, leading to increased crop productivity. The significant interaction effect observed between location and lime application rate (LOC*LM) further confirms that appropriate lime application rates, adjusted according to site conditions, are reflected in key crop response indicators.

## Crop response to treatments

Significant differences (*P* < 0.05) were observed among treatments for key agronomic parameters, including above-ground dry biomass (BMS), grain yield (GY), and harvest index (HI). The highest above-ground dry biomass yield (9,259.2 kg ha^−^^1^) was recorded in the LM3_P1 treatment (Table 2), which combined no lime application (LM3) with the recommended inorganic P rate (69 kg P_2_O_5_ ha^−^^1^ *via* TSP). Comparable biomass yields (8,888.9 kg ha^−^^1^) were observed in LM1_P1 (full lime + TSP) and LM2_P2 (1/4 lime + compost P), suggesting that lime application did not universally enhance biomass production. This may be attributed to the relatively low exchangeable acidity (EA < one cmol kg^−^^1^) at the Holeta and Sademo study sites, where lime requirements were inherently minimal. In such contexts, inorganic P applications (TSP) provided immediate phosphorus availability, driving biomass accumulation, consistent with findings by Tadesse, Alemayehu & Abebe (2019) in similar acidic Ethiopian soils.

**Table 2:** Barley yield and its component as affected by different phosphorus fertilizer and application of soil amendments at Ejere district.

**Level of lime and P sources**		**BMS_KG/HA**	**GY_KG/HA**	**HI (%)**	**TSW**
1	LM1_P1	8,888.9AB	3,300.7ABC	37.8BCD	41.69
2	LM1_P2	6,666.7E	2,478.1D	37.5BCD	40.76
3	LM1_P3	8,101.9BC	2,804.3D	34.27D	42.09
4	LM2_P1	8,240.7ABC	3,387.2AB	41.64A	42.91
5	LM2_P2	7,453.7CDE	2,945.6BCD	40.58AB	42.98
6	LM2_P3	6,851.9DE	2,527.4D	37.16BCD	43.96
7	LM3_P1	9,259.2A	3,478.7A	38.17ABC	43.91
8	LM3_P2	8,240.7ABC	2,902.7CD	35.44CD	43.82
9	LM3_P3	7,870.4BCD	2,928.9BCD	38.46ABC	44.18
LSD (*P* < 0.05)		1,157.2	471.2	3.8	NS
CV %		15.5	16.9	10.8	6.4

**Notes.**

Where: BMS_KG/HA above ground dry biomass in kg/ha; GY_KG/HA mean grain yield in kg/ha; HI harvest index; TSW represent thousand seed weight; LM1, full lime rate based on EA; LM2, 1/4th of lime requirement based on EA; LM3 no lime application; P1, inorganic phosphorus rate; P2, organic/compost; P3, no P fertilizer application. Means with the same latter represent statistically the same; NS not significantly affected by the treatment.

Grain yield followed a similar trend, with the highest value (3,478.7 kg ha^−^^1^) observed in LM3_P1 (no lime + TSP). Treatments LM2_P1 (1/4 lime + TSP) and LM1_P1 (full lime + TSP) produced statistically comparable yields 3,387.2 kg ha^−^^1^ and 3,300.7 kg ha^−^^1^ respectively (Table 2), while the lowest yield 2,478.1 kg ha^−^^1^ occurred in LM1_P2 (full lime + compost P). The poor performance of organic P sources under full lime application may reflect slower mineralization rates of compost-derived phosphorus in limed soils, limiting immediate P availability to crops. This aligns with studies highlighting the delayed nutrient release from organic amendments in high-pH environments (Mamo, Tadesse & Wondimu, 2016; Wondimu, Mamo & Gebremariam, 2021).

Harvest index (HI), a measure of reproductive efficiency, peaked at 41.64% in LM2_P1 (1/4 lime + TSP), followed by LM2_P2 (1/4 lime + compost P) and LM3_P1 (no lime + TSP). The lowest HI was recorded in LM1_P3 (full lime + no P) (Table 2), underscoring the critical role of phosphorus in grain formation. The superior HI in LM2_P1 suggests that moderate lime application (1/4 EA) optimized soil pH for phosphorus uptake without over-suppressing micronutrient availability, a balance previously documented in integrated soil fertility studies (Kiflu, Beyene & Jeff, 2017).

## Soil inorganic phosphorus fractionation

Soil P fractionation revealed distinct shifts in phosphorus pools across treatments (Table 3). Inorganic P sources (TSP) preferentially increased labile phosphorus fractions (Al-P and Fe-P), while compost applications enhanced organic-bound and occluded P. The LM3_P1 treatment, despite lacking lime, showed elevated Ca-P fractions, likely due to inherent soil calcium in Nitisols buffering acidity. Conversely, full lime applications (LM1) reduced Al-P and Fe-P concentrations, consistent with P fixation mechanisms in neutralized soils (Smillie & Syers, 1972). These dynamics highlight the trade-offs between immediate P availability (favored by TSP) and long-term P stabilization (associated with compost and lime), as noted in Ethiopian highland soils by Wondimu, Mamo & Gebremariam (2021).

**Table 3:** Inorganic phosphorus (P_i_) fractions as affected by different soil management/input application P_i_ fractionation results.

Batch		B I	B II	B III	B IV	B V	B VI	
Extractants		1M NH_4_Cl	0.5M NH_4_F	0.1M NaOH	0.3M Na-citrate + Na-dithionite	0.1M NaOH + 1.5M KMnO4	0.25M H2SO4	Total P_i_
No.	TRT	water soluble P	Al-P	Fe-P	R-P	O-P	Ca-P	
1	LM1_P1	5.33	20.07	74.00	21.67	77.33	4.67	203.07
2	LM1_P2	6.00	19.73	97.33	8.67	74.00	6.67	212.40
3	LM1_P3	5.67	22.73	92.67	8.00	72.00	6.67	207.73
4	LM2_P1	3.33	20.40	32.33	25.00	75.67	5.67	162.40
5	LM2_P2	3.33	24.07	47.33	22.00	71.00	6.33	174.07
6	LM2_P3	5.67	26.40	95.00	28.00	77.67	5.67	238.40
7	LM3_P1	5.00	29.07	40.33	31.67	72.00	5.67	183.73
8	LM3_P2	3.33	24.40	113.33	35.67	74.33	6.33	257.40
9	LM3_P3	5.33	31.07	56.67	22.00	66.00	6.67	187.73
Mean		4.8	24.2	72.1	22.5	73.3	6.0	203.0
1	SDM_P_io_	3.67	28.07	94.33	24.67	53.00	7.00	210.73
2	HC_P_io_	6.33	28.40	53.33	41.33	70.00	10.00	209.40
3	DM_P_io_	8.00	42.07	72.00	3.33	84.33	13.00	222.73

**Notes.**

Where: P_io_ is initial composite soil sample, SDM for sample from Sademo, HC from Holeta on-station whereas DM is Damotu PAs located at Ejere district. B I - B VI represents the P pools or baches of extraction as stated in the proceeding rows. All of the units are express P concentration in ppm. Al-P Aluminum-bonded Phosphorus, Fe-P Iron bonded P, R-P Reducible phosphorus, O-P Occluded phosphorus, Ca-P Calcium-Bonded Phosphorus, P_i_ total inorganic P.

The results suggest that in moderately acidic soils (EA < one cmol kg^−^^1^), lime application may not be essential for maximizing short-term crop productivity when using soluble P fertilizers like TSP. However, integrated approaches combining reduced lime rates (*e.g.*, 1/4 EA) with inorganic P could balance pH adjustment and nutrient availability. Organic P sources, while beneficial for soil health, may require complementary strategies (*e.g.*, pre-composting or split applications) to synchronize mineralization with crop demand. These findings align with broader recommendations for context-specific soil fertility management in phosphorus-deficient agroecosystems (Kihara et al., 2016; Sileshi et al., 2019). Tables 1 and 2. Barley crop response data for phosphorus fractionation field trial at 2022 HARC (Welmera and Ejere districts) over location ANOVA result.

*The total inorganic phosphorus* (*P*_*i*_): content in the study areas was found to be 210.73 ppm at Sademo, 209.4 ppm at Holeta, and 222.7 ppm at Damotu. These variations in P_i_ levels are likely influenced by the physicochemical properties of the soils, particularly the presence of iron-bound phosphorus (Fe-P) and occluded phosphorus, which were observed in higher fractions. The significant impact of Fe-P and occluded phosphorus on total P_i_ can be attributed to their roles as major reservoirs of phosphorus in soils (Chang & Jackson, 1957; Hedley, Stewart & Chauhan, 1982). The higher fractions of Fe-P and occluded phosphorus suggest that these forms of phosphorus are dominant in the study areas, potentially due to the specific soil conditions that favor their formation. For instance, Fe-P is often associated with iron oxides, which are prevalent in soils with certain redox conditions (Bünemann, Oberson & Frossard, 2011). Similarly, occluded phosphorus, which is trapped within soil minerals, can also contribute significantly to the total P_i_ pool (Tiessen & Moir, 1993). Understanding these phosphorus fractions is crucial for managing soil fertility and optimizing phosphorus availability for plant growth. Overall, the variations in total P_i_ among the study locations highlight the importance of considering the specific physicochemical properties of each soil when assessing phosphorus dynamics and developing strategies for improving soil fertility.

**Figure 2:**
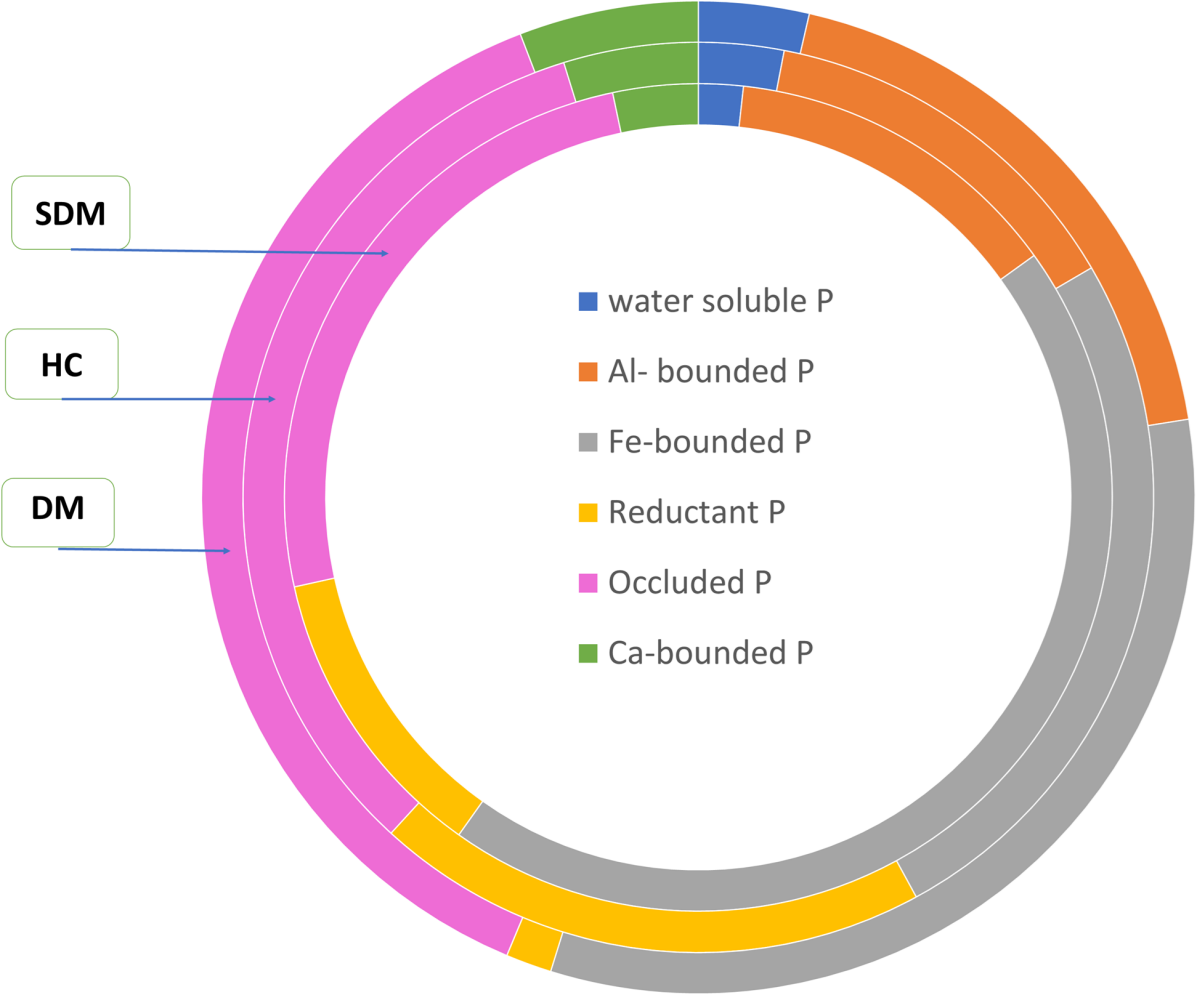
Present share of inorganic phosphorus fractions from initial soil samples the inner for the Damotu, the middle ring for Holeta and the outer ones for the Sademo initial soil sample.

**Figure 3:**
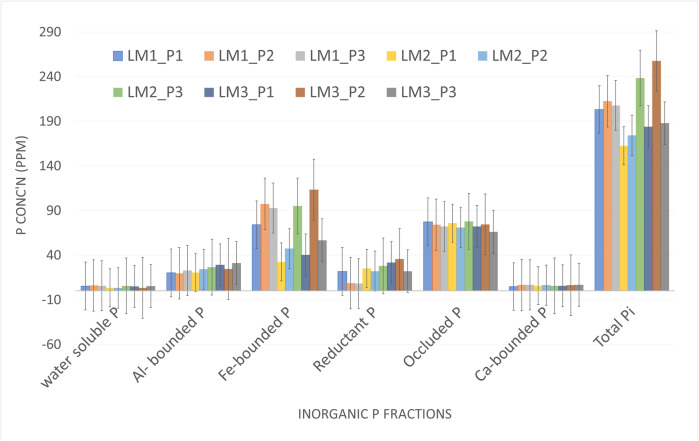
Effects of different soil input treatments on the inorganic phosphorus fractions P_i_ at Sademo study area.

Initial soil inorganic P fractionation results indicated that the highest total P_i_ was observed at the Damotu site, which also had higher water-soluble, Al-bonded, occluded, and Ca-bonded P than the other study sites (Fig. 2) as this result indicated lime amendment could potentially release relatively good proportions of available P to the crop uses whereas the remaining study sites have relatively lower proportion of Al- bonded phosphorus fraction (Fig. 3). The treatment effects on inorganic P_i_ fractions over a single season revealed that it is challenging to significantly alter the entire P_i_ pool through short-term soil amendments. Prolonged application periods are often required to induce measurable changes in phosphorus dynamics, as transformations between different P_i_ fractions depend on soil chemistry and environmental conditions (Hedley, Stewart & Chauhan, 1982). For example, Fe-P and occluded P are highly stable forms that resist rapid transformation under typical agricultural practices.

These results underscore the importance of tailoring soil management strategies to site-specific conditions. Lime amendments may be particularly effective in areas like Damotu with higher Al-P fractions, while other approaches may be needed for sites dominated by Fe-P or occluded P to improve phosphorus availability for crops.

*NH*_4_*Cl extracted P* (*water soluble P*): of the three study area was varied in some extents 1.7, 3.0, 3.6% from the total inorganic P at Sademo, Holeta and Damotu respectively which is quite lower to the plant requirements similar study on acidic soil of southern Ethiopia (Hagere selam) showed very low available forms of phosphorus up to 2% was reported from the total P-pool which negatively affect the plant growth and an indicative of P management requirement to attain effective crop production in such type of soils (Kiflu, Beyene & Jeff, 2017). Likely this phosphorus pool was the one which was affected by the application of different sources of phosphate fertilizer (Amaizah et al., 2012) so application of P fertilizer from different input sources can improve the availability and crop productivity as observed in the crop response results.

*Aluminum-bonded Phosphorus (Al-P):* According to the inorganic phosphorus (P_i_) fractionation results, Al-P constituted approximately 13%, 14%, and 19% of the total P_i_ at the Sademo, Holeta, and Damotu locations, respectively. A similar study reported that Al-P accounted for 16–35% of the total P_i_, representing a significantly higher proportion of inorganic phosphorus (Amaizah et al., 2012). The result from different soils indicates that the higher exchangeable acidity soil also has high Al-P due to higher Al content which aligns with many studies conducted on acidic soils (Gillespie, Antonangelo & Zhang, 2021; Antonangelo et al., 2017; Wang, Wang & Fan, 2015; Ifansyah, 2014; Hsu & Rennie, 1962). Some amendments like liming and organic inputs can improve by substituting Al-P to make it available for crop uses or transform into water soluble P it was observed in the crop response trials (Dejene, Abera & Desalegn, 2023). In acidic mineral soils P is fixed mainly by Al and Fe.

*Iron bonded Phosphorus* (Fe-P): inorganic P fraction which was extracted by NaOH takes the larger P fraction in the P_i_ which ranges in the study area approximately 26, 32 and 45% at Holeta, Damotu and Sademo respectively. A similar study indicate that certain soil minerals can adsorb or co-precipitate with phosphate, and this mineral-bound P provides a potentially large P reservoir in soils. Iron (Fe) oxyhydroxides have a high capacity to adsorb phosphate (Herndon et al., 2020). This pool of P_i_ was less likely affected by single season inorganic/organic phosphorus fertilizer application instead it was affected by the mineralogy and weathering status of the soil. As observed in the different study sites, as a major portion of total P_i_ long-term management related to Fe-P is critical to improve soil extractable P for crop utilization.

*Reducible phosphorus (R-P)*: In the study area, this P_i_ fraction ranged from approximately 1% to 19% across the different sites. However, no consistent result in R-P levels could be observed within a single growing season in response to fertilizer and soil amendment treatments. In P fractionation studies, the R-P fraction generally represents phosphorus associated with Fe and Al oxides and hydroxides. This form of phosphorus is extracted using reducing agents like sodium dithionite, which dissolve Fe and Al oxides and release the bound phosphorus.

R-P is considered moderately labile less immediately available to plants than more labile P forms, but potentially accessible under specific conditions, such as in waterlogged or reducing environments (Wierzbowska et al., 2020). Its availability is influenced by several soil properties, including pH, redox potential, organic matter content, and microbial activity, all of which affect the solubility of Fe and Al compounds (Jin et al., 2021). Although R-P may not contribute significantly to plant-available phosphorus under normal aerobic conditions, it can act as a reserve pool of P that becomes accessible under anaerobic or reducing conditions. Therefore, understanding the dynamics of the R-P fraction is essential for optimizing fertilizer management, especially in soils where Fe and Al oxides strongly bind phosphorus and limit its immediate availability (Wang, Zhang & Müller, 2023)

*Occluded phosphorus (O-P*): The results showed that O-P fraction of P_i_ accounted for 25% at Sademo, 33% at Holeta, and 38% at Damotu, indicating a significant portion of phosphorus that is not readily available for immediate plant uptake. This fraction represents phosphorus that has been encapsulated or bound within secondary minerals like iron (Fe) and aluminum (Al) oxides during soil formation, making it inaccessible under normal conditions. Occluded phosphorus is considered a “sink” in soil phosphorus dynamics, as it is locked away within mineral matrices. Studies have shown that occluded P can constitute a substantial portion of total inorganic P, ranging from 26% to 68% in soils formed over different parent materials (Ohaeri & Eshett, 2011). This aligns with the results from Sademo, Holeta, and Damotu, where occluded P accounts for a notable fraction of total P. The high percentage at Damotu (38%) may indicate advanced weathering or soil development stages, as occlusion increases over time due to pedogenic processes. The formation of occluded P is influenced by soil properties such as clay content, pH, and the presence of Fe and Al oxides. Research shows that occluded P correlates positively with clay and exchangeable calcium but negatively with sand and pH. This suggests that soils with higher clay content or lower pH may favor the accumulation of O-P, which could explain site-specific variations among Sademo, Holeta, and Damotu.

While O-P is not directly available to plants, it represents a long-term reservoir of phosphorus that might be released under specific conditions (*e.g.*, reduced environments or microbial activity). Techniques such as phosphate-solubilizing microbial interactions have been proposed to mobilize occluded P for plant use (Zhu et al., 2024). However, its high proportion in soils highlights the need for targeted management practices to enhance phosphorus availability for crops.

*Calcium-Bonded Phosphorus (Ca-P)*: These proportion of P-pool in the total inorganic phosphorus (P_i_) was found to be approximately 3% at Sademo, 5% at Holeta, and 6% at Damotu. Notably, the application of different organic and inorganic soil amendments over a single season did not significantly impact this phosphorus fraction, (Fig. 2). This result is consistent with existing studies, which suggests that Ca-P levels are generally low in acidic soils (Chang & Jackson, 1957; Hedley, Stewart & Chauhan, 1982). The study area’s soil pH, which ranged from strong to very strong acidity, supports this observation.

The limited presence of Ca-P in these acidic soils can be attributed to the reduced availability of calcium ions for phosphorus binding under such conditions (Bünemann, Oberson & Frossard, 2011). As noted by Tiessen & Moir (1993), calcium-bound phosphorus is typically more prevalent in alkaline soils where calcium carbonate is more soluble. In contrast, the acidic environment in our study areas would have limited the formation and stability of Ca-P, making it less responsive to the applied amendments. Furthermore, the lack of significant impact from soil amendments on Ca-P levels suggests that these amendments did not alter the soil pH sufficiently to affect the solubility and availability of calcium for phosphorus binding. This highlights the importance of considering soil pH when evaluating phosphorus dynamics and the potential effectiveness of different soil amendments in enhancing phosphorus availability.

## Conclusion and Recommendation

The study provides critical insights into phosphorus fractionation dynamics across contrasting soil types in Ethiopia, emphasizing the need for soil-specific management strategies. Lime amendments demonstrated notable efficacy in strongly acidic soils by mobilizing bonded P fractions, thereby enhancing crop availability. However, moderately acidic soils with inherently low available P require innovative strategies beyond liming, such as organic amendments or microbial interventions, to unlock recalcitrant P pools like Fe-bonded and occluded fractions. The observed variability in P fraction responses underscores the importance of tailoring amendments to local soil characteristics, offering a pathway to optimize fertilizer efficiency and reduce environmental losses. These findings pioneer a framework for sustainable P management in diverse agroecosystems. Accordingly, the following major recommendation were made.

Prioritizing lime application on strongly acidic soils is critical actively promote lime application in regions with severe soil acidity (pH < 5.5 and EA < one cmol kg^−^^1^) to mitigate Al/Fe toxicity and solubilize fixed P, directly boosting crop yields.

Combine organic fertilizers (*e.g.*, compost, manure) with microbial inoculants or redox-enhancing practices to target Fe-bound, Reductant P and occluded P in soils with moderately acidic soils (pH 5.5–6.5) might address limitations of lime alone which needs to be confirmed in further studies.

Conducting multi-season studies to evaluate synergistic effects of lime, organic matter, and phosphorus-solubilizing microorganisms on P fraction stability and crop uptake is essential.

Develop region-specific P management protocols based on soil texture, mineralogy, and initial P fraction profiles to maximize resource-use efficiency. Additionally, it is important to expand research on P fraction distribution across Ethiopia’s major agroecological zones to inform large-scale precision fertilization campaigns.

By aligning amendment strategies with soil-specific P fraction dynamics, policymakers and farmers can co-design resilient nutrient management systems that bridge productivity gaps while safeguarding soil health for future generations.

## Limitations of the Study and Future Directions

A key limitation of this study is the need to extend experiments to soils with stronger acidity and to conduct multi-season trials. This is essential to more accurately capture the long-term effects of organic fertilizer sources and their prolonged application on the less labile or inner phosphorus pools, which are not easily altered by amendments such as agricultural lime.

Additionally, it is critical to evaluate phosphorus fractionation across diverse soil types, considering their distinct physico-chemical properties, to better understand how these properties influence phosphorus mobilization into plant-available forms. Such studies will provide a comprehensive understanding of soil-specific phosphorus dynamics.

Future research should also incorporate the use of efficient phosphate solubilizing microorganisms (PSMs). These beneficial bacteria and fungi play a vital role in converting insoluble phosphorus forms into bioavailable ones, enhancing phosphorus uptake and promoting sustainable crop production. Exploring their application could lead to eco-friendly strategies for improving phosphorus use efficiency, particularly in challenging soil conditions.

## Supplemental Information

10.7717/peerj.20410/supp-1 Supplemental Information 1 Raw data for phosphorus fractination

10.7717/peerj.20410/supp-2 Supplemental Information 2 Raw data of crop response

10.7717/peerj.20410/supp-3 Supplemental Information 3 Phosphorus fractionation study at field and laboratory condition

## Additional Information and Declarations

### Data Availability

The following information was supplied regarding data availability: The raw data is available in the Supplemental Files.
