# Supplementary material for: Phosphorus fractionation and distribution in Nitisols of the central Ethiopian highlands affects soil nutrient management strategies in barley (Hordeum vulgare L.) production
Source: PeerJ. 2025 Dec 19;13:e20410. doi: 10.7717/peerj.20410 (PMC12721123; doi:10.7717/peerj.20410)
Supplement: Supplemental Information 3 [file peerj-13-20410-s003.docx]

| 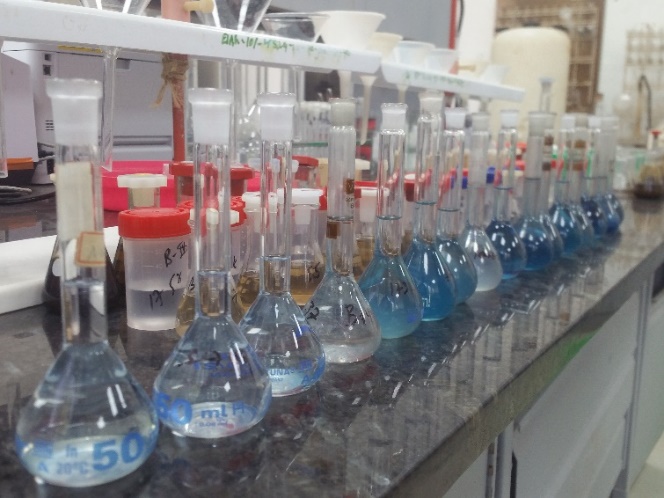 | 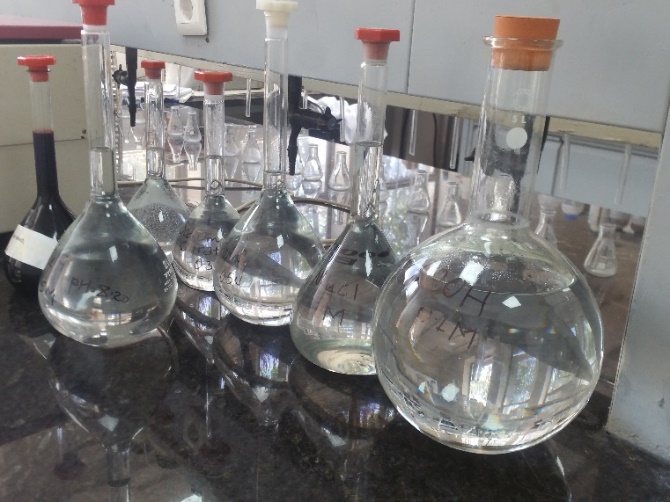 |
| --- | --- |
| Batch-I Samples varied color intensity at reading | Prepared extractants for sequential P extraction |
| 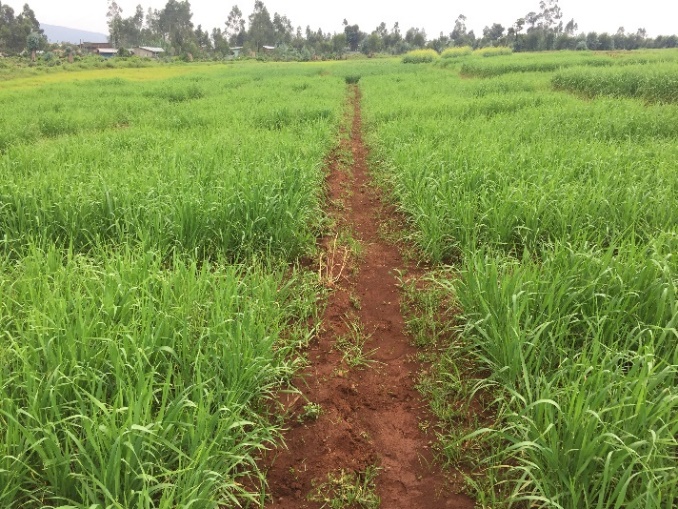 | 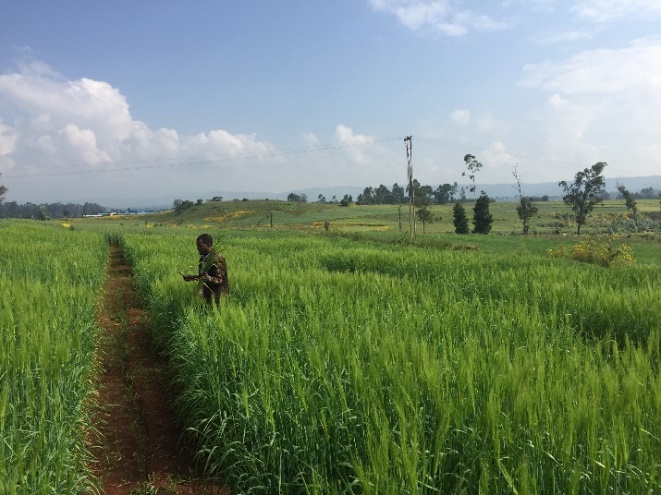 |
| 3^rd^ week after traement application at Damotu | Tissue sample collection at booting, Holeta |

**Supplementary figure 1**. Phosphorus fractionation study at field and laboratory condition.
